# Dual spike and nucleocapsid mRNA vaccination confer protection against SARS-CoV-2 Omicron and Delta variants in preclinical models

**DOI:** 10.1126/scitranslmed.abq1945

**Published:** 2022-09-14

**Authors:** Renee L. Hajnik, Jessica A. Plante, Yuejin Liang, Mohamad-Gabriel Alameh, Jinyi Tang, Srinivasa Reddy Bonam, Chaojie Zhong, Awadalkareem Adam, Dionna Scharton, Grace H. Rafael, Yang Liu, Nicholas C. Hazell, Jiaren Sun, Lynn Soong, Pei-Yong Shi, Tian Wang, David H. Walker, Jie Sun, Drew Weissman, Scott C. Weaver, Kenneth S. Plante, Haitao Hu

**Affiliations:** 1 Department of Microbiology and Immunology, University of Texas Medical Branch, Galveston, TX 77555, USA.; 2 Department of Pathology, University of Texas Medical Branch, Galveston, TX 77555, USA.; 3 Institute for Human Infections and Immunity, University of Texas Medical Branch, Galveston, TX 77555, USA.; 4 World Reference Center for Emerging Viruses and Arboviruses, University of Texas Medical Branch, Galveston, TX 77555, USA.; 5 Department of Medicine, University of Pennsylvania Perelman School of Medicine, Philadelphia, PA 19104, USA.; 6 Division of Pulmonary and Critical Medicine, Department of Medicine, Mayo Clinic, Rochester, MN 55905, USA.; 7 Carter Immunology Center, University of Virginia, Charlottesville, VA 22908, USA.; 8 Division of Infectious Disease and International Health, Department of Medicine, University of Virginia, Charlottesville, VA 22908, USA.; 9 Department of Biochemistry and Molecular Biology, University of Texas Medical Branch, Galveston, TX 77555, USA.; 10 Sealy Institute for Vaccine Sciences, University of Texas Medical Branch, Galveston, TX 77555, USA.; 11 Center for Biodefense and Emerging Infectious Diseases, University of Texas Medical Branch, Galveston, TX 77555, USA. Diseases, University of Texas Medical Branch, Galveston, TX 77555, USA.

## Abstract

Emergence of SARS-CoV-2 variants of concern (VOCs), including the highly transmissible Omicron and Delta strains, has posed constant challenges to the current COVID-19 vaccines that principally target the viral spike protein (S). Here, we report a nucleoside-modified messenger RNA (mRNA) vaccine that expresses the more conserved viral nucleoprotein (mRNA-N) and show that mRNA-N vaccination alone can induce modest control of SARS-CoV-2. Critically, combining mRNA-N with the clinically proven S-expressing mRNA vaccine (mRNA-S+N) induced robust protection against both Delta and Omicron variants. In the hamster models of SARS-CoV-2 VOC challenge, we demonstrated that, compared to mRNA-S alone, combination mRNA-S+N vaccination not only induced more robust control of the Delta and Omicron variants in the lungs but also provided enhanced protection in the upper respiratory tract. In vivo CD8^+^ T cell depletion suggested a potential role for CD8^+^ T cells in protection conferred by mRNA-S+N vaccination. Antigen-specific immune analyses indicated that N-specific immunity, as well as augmented S-specific immunity, was associated with enhanced protection elicited by the combination mRNA vaccination. Our findings suggest that combined mRNA-S+N vaccination is an effective approach for promoting broad protection against SARS-CoV-2 variants.

## INTRODUCTION

Coronavirus disease 2019 (COVID-19), caused by severe acute respiratory syndrome coronavirus 2 (SARS-CoV-2), has spread rapidly and led to a major pandemic since its detection in December 2019 (1, 2). A number of vaccines based on various platforms have been developed in response to the COVID-19 pandemic (3), among which two mRNA vaccines and the adenovirus type 26–vectored vaccine showed high efficacy in late-stage clinical trials and have been licensed or received emergency use authorization in the United States and many other regions of the world (3). Although the rapid development of these first-generation vaccines provides hope for ending the COVID-19 pandemic, the emergence of variants of concern (VOCs), including the highly transmissible Delta and Omicron strains, has posed constant challenges to vaccine-induced immunity (4–11). Multiple spike (S) protein variants have been identified with reduced sensitivity to vaccine-induced neutralization (6–8). Clinical studies also indicate that current COVID-19 vaccines show decreased efficacy against the Delta (12) and Omicron (13) variants.

Current COVID-19 vaccines principally target the viral S protein or its receptor binding domain, with the major goal of eliciting a potent neutralizing antibody response (3). We hypothesize that a vaccine approach targeting a more conserved viral protein, in addition to S, would provide broader protection against VOCs. Among the SARS-CoV-2 viral proteins, the nucleoprotein (N) is a major structural protein involved in viral assembly and genome packaging (14). Bioinformatic analysis showed that, compared to S, the N protein is more conserved between SARS-CoV-1 and SARS-CoV-2 (90 and 76% amino acid sequence identity for N and S, respectively) and across different coronaviruses (15–17). Immunologically, N is an immunodominant protein and triggers a strong T cell response that has been shown to correlate with viral control in SARS-CoV-2 infection (18, 19). Immune analysis of SARS-CoV-1–infected individuals showed that N-specific memory T cells remained detectable 17 years after the 2002 to 2003 SARS outbreak and manifested robust cross-reactivity with the N protein of SARS-CoV-2 (20), indicating that N-specific T cell immunity is long-lasting and may provide broader protection against various coronaviruses (21). Thus, the N protein is considered a promising immunogen for incorporation in SARS-CoV-2 vaccine designs (22, 23) to generate a more robust and broadly protective vaccine platform in the face of future viral mutations.

In this study, we generated a nucleoside-modified (m1Ψ) mRNA vaccine that encodes the full-length N protein of SARS-CoV-2 (Wuhan-Hu-1 strain) and formulated it in lipid nanoparticles (LNPs). We showed that mRNA-N was highly immunogenic and induced robust N-specific T cell responses and binding antibodies. In mouse and hamster challenge models, we demonstrated that mRNA-N alone induced modest protection against SARS-CoV-2. Furthermore, we combined mRNA-N with the clinically proven S mRNA vaccine (mRNA-S) (24, 25) and tested its protective efficacy against SARS-CoV-2 Delta and Omicron variants. Our data showed that combination mRNA vaccination induced more robust control of the Delta and Omicron variants in the lungs and also provided additional control of both variants in the upper respiratory tract. Our study suggests potential broad utility of our mRNA vaccine approach against current and emerging SARS-CoV-2 VOCs.

## RESULTS

### mRNA-N vaccine is immunogenic and induces robust antibody and T cell response in mice

The coronavirus N protein is an important viral antigen that induces durable and broadly reactive T cells. We generated a methyl-pseudouridine–modified (m1Ψ) mRNA that encodes full-length N protein (QHD43423.2) of the ancestral, or wild-type, SARS-CoV-2 strain (Wuhan-Hu-1; fig. S1A). Synthesis, purification, and LNP formation of the mRNA-N vaccine were conducted as previously described (26–28). Western blot analysis confirmed the expression of the N protein in 293T cells after mRNA-N-LNP treatment (fig. S1B).

**Fig. 1.**
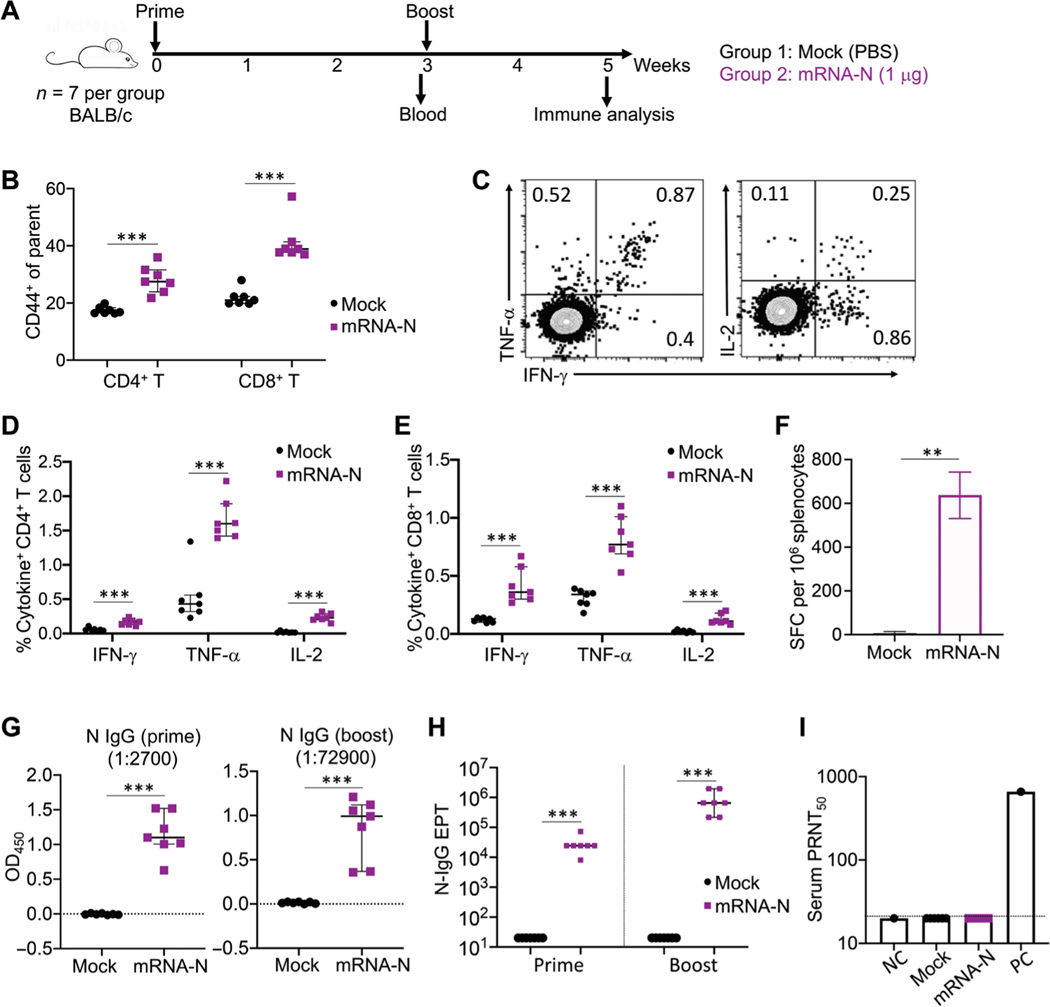
mRNA-N vaccination is immunogenic in mice. (**A**) Experimental design and timeline. Two groups of BALB/c mice (*n* = 7) were intramuscularly vaccinated with PBS (Mock) or mRNA-N vaccine (1 μg) at weeks 0 and 3. At week 3 before booster vaccination, blood and serum samples were collected for analysis of antibody response. Two weeks after booster vaccination (week 5), mice were euthanized and subjected to immune analysis. (**B**) Analysis of total CD4+ and CD8+ T cell activation in the mouse spleen at week 5 after immunization. Expression of CD44 on CD4+ and CD8+ T cells was examined by flow cytometry and shown as percent CD44+ of parental population. (**C**) Vaccine-specific T cells in mouse spleen were measured by ICS. Splenocytes were stimulated with a SARS-CoV-2 N peptide pool (QHD43423.2), followed by immune staining and flow cytometric analysis. Representative flow cytometry plots for cytokine expression in T cells are shown. (**D**) Shown is the comparison of percent cytokine-positive, N-specific CD4+ T cells in the spleen between mock and vaccine groups. (**E**) Shown is the comparison of percent cytokine-positive, N-specific CD8+ T cells in the spleen between mock and vaccine groups. (**F**) N-specific T cells in the spleen were measured by IFN-γ ELISPOT. Data were shown as SFC per 106 splenocytes. (**G**) ELISA measurements are shown for serum N-specific–binding IgG after prime (week 3) or booster (week 5) vaccination. Optical density (OD_450_) values for individual serum samples after prime or booster vaccination at indicated serum dilution (1:2700 for prime; 1:72900 for booster) are shown. (**H**) Comparison of N-specific IgG end point titers (EPT) between mock and vaccine groups after prime and booster vaccination is shown. (**I**) Serum neutralizing activity was measured by plaque reduction neutralization test (PRNT) using wild-type SARS-CoV-2. PRNT_50_ for individual serum samples of the mock and vaccine groups are shown. Dashed line in (I) indicates the limit of detection. NC, negative control; PC, positive control. Data are presented as median and IQR. Mann-Whitney (F to H) or Kruskal-Wallis (B to E) test was used for statistical analysis. **P < 0.01 and ***P < 0.001.

The immunogenicity of mRNA-N was evaluated in BALB/c mice (Fig. 1A). Two groups of mice (*n* = 7 per group) were vaccinated with phosphate-buffered saline (PBS; mock) or mRNA-N (1 μg), a dose selected on the basis of previous studies testing similar mRNA-LNP in mice (29). Vaccination was given intra-muscularly at week 0 (prime) and week 3 (boost). Three weeks after prime vaccination (on the day of the booster), serum samples were collected for analysis of antibody responses; two weeks after the booster (week 5), mice were euthanized and subjected to immunological analyses (Fig. 1A). First, T cell responses were examined in splenocytes by flow cytometry. On the basis of CD44 expression, a marker used for T cell activation and memory (30, 31), we observed that, compared to the mock controls, mRNA-N induced strong CD4^+^ and CD8^+^ T cell activation in the spleen (Fig. 1B). The N-specific T cell response was measured by intracellular cytokine staining (ICS) after stimulation of splenocytes with a 15-amino acid peptide pool that spans the entire N protein (QHD43423.2). Representative flow cytometry plots for cytokine expression in T cells are shown in Fig. 1C and fig. S2A. Compared to the mock controls, mRNA-N induced high N-specific CD4^+^ and CD8^+^ T cell responses (*P* < 0.001 for all three cytokines; Fig. 1, D and E). N-specific T cells appeared to predominantly express tumor necrosis factor–α (TNF-α; median, 1.60% for CD4^+^ T cells and 0.77% for CD8^+^ T cells), followed by interferon-γ (IFN-γ) and interleukin-2 (IL-2; Fig. 1, D and E). The mRNA-N vaccine–induced T cell response was also evaluated by an IFN-γ enzyme-linked immunosorbent spot (ELISPOT) assay (fig. S2B). Compared to mock controls, the vaccine elicited high numbers of N-specific T cells in the spleen [median spot-forming cells (SFC)/10^6^ splenocytes for mock versus mRNA-N: 6 versus 638; *P* < 0.01; Fig. 1F].

We next examined antibodies in the mouse serum samples after prime and booster immunization. Compared to the mock controls, prime immunization induced strong N-specific–binding immunoglobulin G (IgG) responses (Fig. 1G, left), which were further enhanced by the booster (Fig. 1G, right). To determine antibody end point titers (EPTs), serum samples were serially diluted, and N-specific–binding IgG was examined by enzyme-linked immunosorbent assay (ELISA) (fig. S2C). The analysis showed that median IgG EPTs after prime and booster vaccinations were 24,300 and 656,100, respectively (Fig. 1H). Last, serum neutralizing activity was determined by the plaque reduction neutralization test (PRNT). As expected, on the basis of the lack of exposure to the S protein that mediates viral entry, no neutralizing activity was detected in any of the vaccinated animals (Fig. 1I). Together, these data suggest that the mRNA-N vaccine is highly immunogenic and intramuscular immunization induces a robust N-specific T cell immunity and binding antibody response.

### mRNA-N vaccination alone induces modest control of SARS-CoV-2 infection in mice and hamsters

**Fig. 2.**
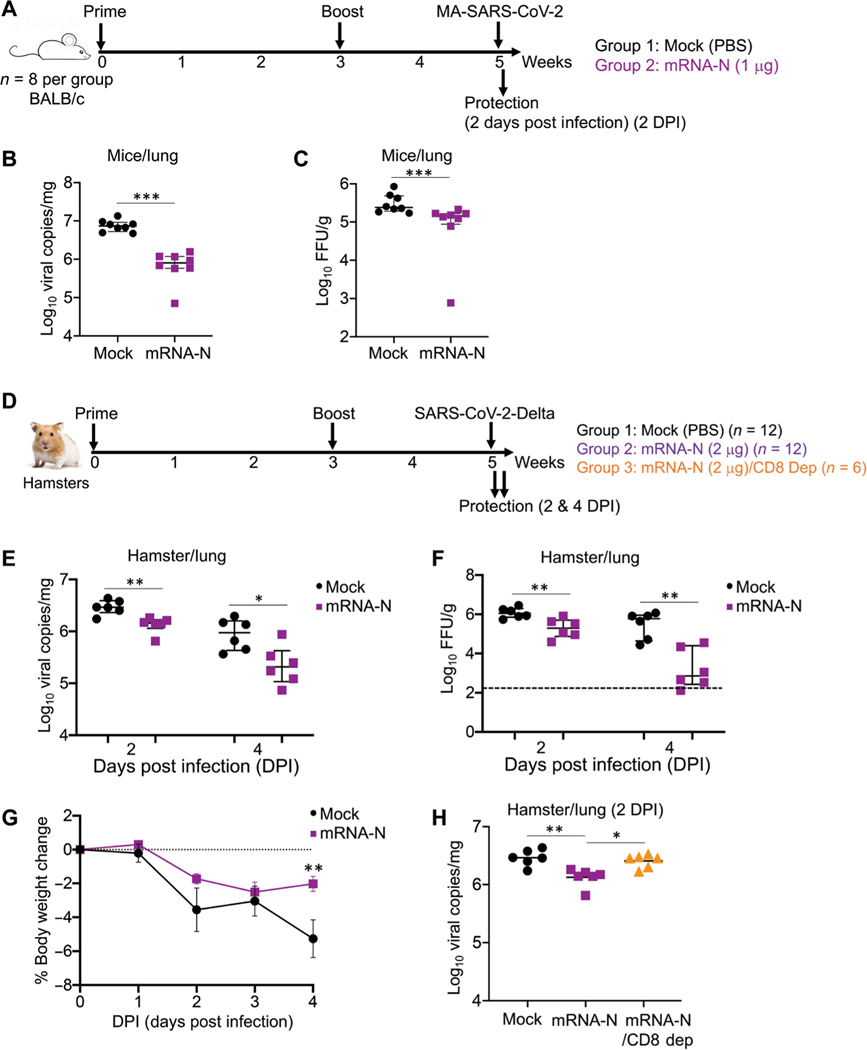
mRNA-N vaccination induced protection against SARS-CoV-2 challenge in mice and hamsters. (**A**) Mouse experimental design and timeline. Two groups of BALB/c mice (*n* = 8) were intramuscularly vaccinated with PBS (mock) or mRNA-N vaccine (1 μg) at weeks 0 and 3. Two weeks after booster vaccination (week 5), mice were intranasally challenged with mouse-adapted (MA) SARS-CoV-2 (2 × 104 pfu). Two days post infection (DPI), viral loads in the lungs were analyzed to evaluate vaccine-induced protection. (**B**) Comparison of viral RNA copies in the mouse lungs between mock and vaccine groups are shown. Viral RNA copies were quantified by RT-PCR and expressed as log_10_ copies per milligram of lung tissue. (**C**) Comparison of viral titers in the mouse lungs between mock and vaccine group are shown. Viral titers were quantified by plaque assay and expressed as log_10_ FFU per gram of lung tissue. (**D**) Hamster experimental design and timeline. Three groups of hamsters were investigated. The first two groups (*n* = 12 per group) were intramuscularly vaccinated with mock or mRNA-N (2 μg) at weeks 0 and 3, followed by SARS-CoV-2 Delta challenge at week 5 and viral load analysis on 2 (*n* = 6) and 4 (*n* = 6) DPI. The third group (*n* = 6) received the same mRNA-N vaccine and subsequent viral challenge, except that these hamsters were intraperitoneally injected with two doses of antibodies for CD8+ T cell depletion at 6 and 3 days before viral challenge. Viral loads were analyzed on 2 DPI (*n* = 6).(**E**) Comparison of viral RNA copies in hamster lungs (log_10_ viral copies per milligram) between mock and vaccine group are shown for samples collected on 2 and 4 DPI. (**F**) Comparisons of viral titers in the hamster lungs (log_10_ FFU per gram) between mock and vaccine group are shown for samples collected on 2 and 4 DPI. (**G**) Comparison of hamster body weight loss is shown for the mock and vaccine group from days 0 to 4 DPI. (**H**) A comparison of viral RNA copies in the lung of hamsters (log_10_ viral copies per milligram) among the three groups is shown for samples collected on 2 DPI. The dashed line in (F) indicates the limit of detection. Data are presented as median and IQR where appropriate. Mann-Whitney (B, C, and G) or Kruskal-Wallis (E, F, and H) test was used for statistical analysis. *P < 0.05, **P < 0.01, and ***P < 0.001.

It remained unclear whether immunization with the N-expressing vaccine alone would induce immune-mediated control of SARS-CoV-2 infection. We evaluated effectiveness of mRNA-N in animal models. First, two groups of BALB/c mice (*n* = 8 per group) were vaccinated with either PBS (mock) or mRNA-N vaccine at week 0 (prime) and week 3 (boost), followed by intranasal challenge with a mouse-adapted SARS-CoV-2 strain [MA-SARS-CoV-2; 2 × 10^4^ plaque-forming units (pfu)] (32) at week 5 (Fig. 2A). Two days post infection (DPI), viral loads in the lungs were quantified for viral RNA and infectious titers. Compared to the mock-immunized controls, intramuscular immunization with mRNA-N induced a modest but significant reduction in viral RNA copies (*P* < 0.001; Fig. 2B) and viral titers (*P* < 0.001; Fig. 2C). We also evaluated the vaccine’s protective effect in mice after intranasal immunization using the same vaccination schedule and challenge dose as in the intramuscular route. In contrast to intramuscular vaccination, intranasal vaccination did not induce notable viral control in the lungs (fig. S3, A and B). Consistent with the lack of protection, no antibody response (binding IgG) was induced in serum samples after mRNA-N intranasal immunization (fig. S3C). Thus, intramuscular immunization was used in all subsequent experiments.

Next, we evaluated the vaccine against the SARS-CoV-2 Delta variant in hamsters, which are susceptible to wild-type SARS-CoV-2 strains. Three cohorts were investigated (Fig. 2D). The first two (*n* = 12 per group) were intramuscularly vaccinated with PBS (mock) or the mRNA-N vaccine (2 μg) at weeks 0 and 3, followed by intranasal challenge with the SARS-CoV-2 Delta strain (2 × 10^4^ pfu). A higher vaccine dose (2 μg) was selected on the basis of previous studies evaluating similar mRNA-LNP COVID-19 vaccines in hamsters (33, 34). For each group, on 2 DPI (*n* = 6) and 4 DPI (*n* = 6), viral RNA copies and infectious titers in the lungs were assayed, along with viral RNA copies in nasal washes. In addition, a third group (*n* = 6) that received the mRNA-N vaccine but underwent in vivo CD8^+^ T cell depletion was included (Fig. 2D). These hamsters received two doses of CD8-depletion antibody (35, 36) at 6 and 3 days before challenge, and viral RNA copies in the lungs were analyzed on 2 DPI (*n* = 6). The data showed that on 2 DPI, compared to the mock control, mRNA-N induced a statistically significant but modest impact on viral RNA copies and infectious titers (*P* < 0.01; Fig. 2, E and F). On 4 DPI, compared to the mock controls, a more profound effect was observed for the vaccine in reducing viral RNA copies (3-fold reduction; *P* < 0.05; Fig. 2E) and infectious titers (173-fold reduction; *P* < 0.01; Fig. 2F).

Hamster body weights were monitored on the day of challenge through 4 DPI. Infection with Delta led to considerable weight loss in the mock-vaccinated group (greater than 5% on 4 DPI); mRNA-N–vaccinated hamsters exhibited reduced weight loss at 4 DPI as compared to mock-vaccinated hamsters (*P* < 0.05; Fig. 2G). These data indicated modest protection conferred by mRNA-N vaccine against SARS-CoV-2 Delta challenge. In addition, we compared viral RNA copies in the lungs of hamsters that received mRNA-N vaccine with or without CD8^+^ T cell depletion (Fig. 2H). CD8^+^ T cell depletion largely abrogated the effect of the vaccine on viral control (Fig. 2H), indicating a potential role for CD8^+^ T cells in mRNA-N–induced protection against SARS-CoV-2 Delta challenge. Last, compared to the mock controls, mRNA-N vaccination did not reduce viral loads in the nasal washes (fig. S3D). These results indicate that mRNA-N alone has minimal impact in the upper respiratory tract, likely because of the vaccine’s inability to induce neutralizing antibodies.

### Combination mRNA vaccination induces robust protection against challenge with MA-SARS-CoV-2 in mice and the SARS-CoV-2 Delta variant in hamsters

**Fig. 3.**
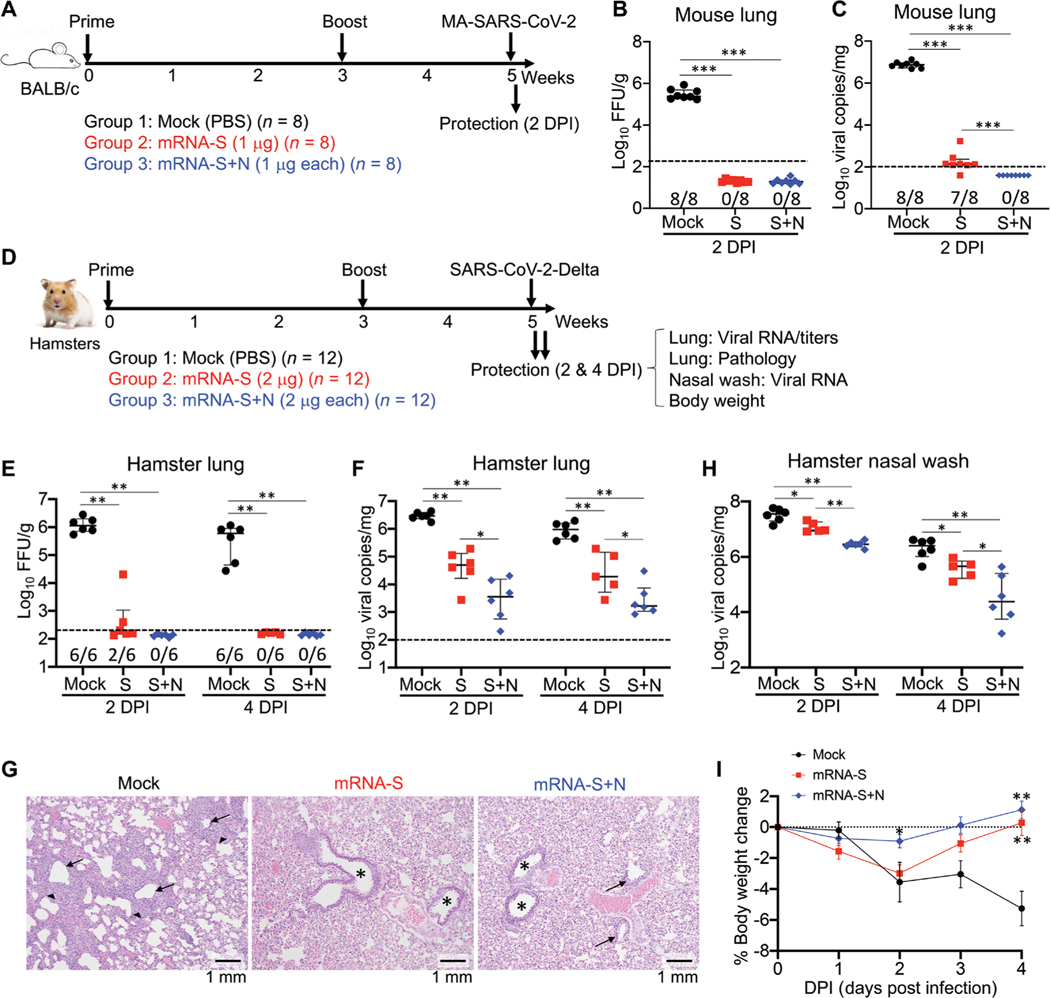
Combination mRNA-S+N vaccination confers improved protection against challenge with the SARS-CoV-2 Delta variant compared to mRNA-S vaccination alone. (**A**) Mouse experimental design and timeline. Three groups of mice (*n* = 8 per group) were vaccinated intramuscularly with mock, mRNA-S (1 μg), or mRNA-S+N (1 μg for each) at weeks 0 and 3, followed by intranasal challenge with MA-SARS-CoV-2 (2 × 104 pfu). On 2 DPI, viral RNA copies and titers in the lungs were quantified. (**B**) A comparison of viral titers between different groups is shown for mouse lungs collected on 2 DPI (log_10_ FFU per gram). (**C**) Shown is a comparison of viral RNA copies in the mouse lungs (log_10_ viral copies per milligram) between different groups at 2 DPI. (**D**) Hamster experimental design and timeline. Three groups of hamsters (*n* = 12 per group) were vaccinated intramuscularly with mock, mRNA-S (2 μg), or mRNA-S+N (2 μg for each) at weeks 0 and 3, followed by intranasal challenge with SARS-CoV-2 Delta strain (2 × 104 pfu) at week 5. On 2 (*n* = 6) and 4 DPI (*n* = 6), lung tissues were harvested for analysis of viral RNA copies, viral titers, and pathology; nasal washes were collected for analysis of viral RNA copies; hamster body weights were also monitored. (**E**) Shown is a comparison of viral titers (log_10_ FFU per gram) in the hamster lungs between different groups on 2 and 4 DPI. (**F**) A comparison of viral RNA copies in the hamster lungs (log_10_ viral copies per milligram) is shown between different groups using samples collected at 2 and 4 DPI. (**G**) Hamster lung histopathology is shown. Postchallenge lung tissues (2 DPI) were fixed, and 5-μm sections were cut from hamsters and stained with hematoxylin and eosin. Left, lung of mock-immunized hamsters demonstrates bronchi with bronchiolitis (arrows) and adjacent marked interstitial pneumonia (arrowheads); middle and right, lungs of hamsters immunized with mRNA-S (middle) or mRNA-S+N (right) demonstrate normal bronchial (stars), bronchiolar (arrows), and alveolar architecture. Scale bars, 1 mm. (**H**) A comparison of viral RNA copies in the nasal washes (log_10_ viral copies per milligram) is shown between the indicated groups on 2 and 4 DPI. (**I**) A comparison of hamster body weight changes is shown between different groups from 0 to 4 DPI. * on 2 DPI denotes difference of mRNA-S+N from mRNA-S or mock; ** on 4 DPI denote differences of mRNA-S+N or mRNA-S from mock, respectively. Dashed lines in (B, C, E, and F) show the assay limit of detection. The numbers at the bottom of (B, C, and E) indicate the proportion of animals with a result above the limit of detection. Data are presented as median and IQR where appropriate. Mann-Whitney (B and C) or Kruskal-Wallis (E, F, H, and I) test was used for statistical analysis. *P < 0.05, **P < 0.01, and ***P < 0.001.

After demonstrating that mRNA-N alone was immunogenic and elicited modest efficacy against SARS-CoV-2, we next explored whether a bivalent vaccine consisting of both mRNA-N and the S-expressing mRNA vaccine (mRNA-S) would induce more robust protection against variants. Thus, in addition to mRNA-N, we also generated an m1Ψ-modified mRNA vaccine that expresses the prefusion-stabilized SARS-CoV-2 S protein with two proline mutations (named “mRNA-S-2P”; Wuhan-Hu-1), similar to Pfizer/BioNTech’s BNT162b (37) and Moderna’s mRNA-1273 (38) vaccines. First, we tested these vaccines in BALB/c mice. Three groups of mice (*n* = 8 per group) were immunized with either PBS (mock), mRNA-S (1 μg), or the combination mRNA-S/mRNA-N vaccine (mRNA-S+N; 1 μg of each mRNA) at weeks 0 and 3, followed by intranasal challenge with the MA-SARS-CoV-2 strain (2 × 10^4^ pfu) at week 5; on 2 DPI, viral titers and RNA copies in the lungs were measured (Fig. 3A). Compared to mock vaccination, both mRNA-S alone and the combined mRNA-S+N induced complete viral control with no detectable infectious virus in the lungs (Fig. 3B). However, quantification of viral RNA by the more-sensitive reverse transcription polymerase chain reaction (RT-PCR) approach revealed a significant difference between mRNA-S and mRNA-S+N (*P* < 0.001; Fig. 3C). Compared to the mock control, mRNA-S alone remained highly effective in reducing viral RNA copies in the lungs (seven of eight with weakly detectable RNA and one of eight with no detect- able RNA); however, mRNA-S+N induced complete protection against viral RNA in the lungs in all eight mice tested (Fig. 3C).

Second, we tested these vaccines against the Delta variant in hamsters. Three groups (*n* = 12 per group) were vaccinated with PBS (mock), mRNA-S (2 μg), or mRNA-S+N (2 μg for each) at weeks 0 and 3, followed by intranasal challenge at week 5. On 2 (*n* = 6) and 4 DPI (*n* = 6), hamsters were analyzed for vaccine-induced protection on the basis of viral loads, lung histopathologic lesions, and body weight loss (Fig. 3D). On 2 DPI, compared to mock vaccinations, mRNA-S alone induced substantial control of infectious virus in the lungs (two of six with detectable titers), whereas mRNA-S+N induced complete viral control with no detectable titers in any of the six hamsters (Fig. 3E). On 4 DPI, both mRNA-S and mRNA-S+N vaccinations completely controlled virus in the lungs (Fig. 3E). Analysis of viral RNA revealed a more profound difference between mRNA-S and mRNA-S+N. Compared to mock vaccination, mRNA-S alone reduced lung viral RNA copies by 57-fold (*P* < 0.01; Fig. 3F). Critically, relative to mRNA-S, mRNA-S+N induced a more robust viral control on 2 DPI and reduced viral RNA copies by an additional 12-fold (*P* < 0.05 for mRNA-S versus mRNA-S+N; Fig. 3F). Compared to mock vaccination, mRNA-S+N induced a 770-fold reduction in median viral RNA copies (Fig. 3F). A similar result was observed on 4 DPI (Fig. 3F). Consistent with the viral load data, lung histopathological analysis showed that on 2 DPI, Delta challenge caused evident changes in the mock-immunized hamsters, including bronchiolitis and interstitial pneumonia (Fig. 3G). Hamsters vaccinated with mRNA-S or mRNA-S+N were all protected from these lesions and demonstrated normal bronchial, bronchiolar, and alveolar architecture (Fig. 3G). The data indicate that mRNA-S vaccine itself is effective against disease caused by SARS-CoV-2 Delta, consistent with clinical findings (39) and further show that mRNA-S+N was highly protective in both models.

Viral RNA copies in nasal washes were examined on 2 and 4 DPI. Unlike the robust viral control provided by mRNA-S in the lungs, mRNA-S was less effective in reducing viral RNA copies in the nasal washes on both 2 (mock versus mRNA-S, threefold reduction) and 4 DPI (fivefold reduction) (Fig. 3H). These data suggest that mRNA-S induces strong protection against disease but reduced protection against infection and upper airway shedding by the Delta variant (12). Compared to mRNA-S alone, mRNA-S+N induced more robust viral control in the nasal washes on 2 DPI (mock versus mRNA-S+N, 11-fold reduction; *P* < 0.01) and 4 DPI (98-fold reduction, *P* < 0.05; Fig. 3H). Together, these data support that combination mRNA-S+N vaccination induces stronger and faster control of SARS-CoV-2 Delta infection in both lungs and the upper respiratory tract as compared to mRNA-S alone, indicating that this vaccine approach may also reduce the risk of transmission.

Body weights analysis showed that challenge with the Delta variant caused progressive weight loss in the mock-vaccinated hamsters, declining by greater than 5% on 4 DPI (Fig. 3I), which is comparable with that caused by the wild-type SARS-CoV-2 (40). Compared to mock vaccination, mRNA-S alone or the bivalent mRNA-S+N protected hamsters from weight loss on 3 and 4 DPI (Fig. 3I). Last, compared to mRNA-S alone, mRNA-S+N resulted in significantly reduced weight loss at 2 DPI (*P* < 0.05; Fig. 3I).

### Combination mRNA vaccination induces robust protection against the SARS-CoV-2 Omicron variant in hamsters

**Fig. 4.**
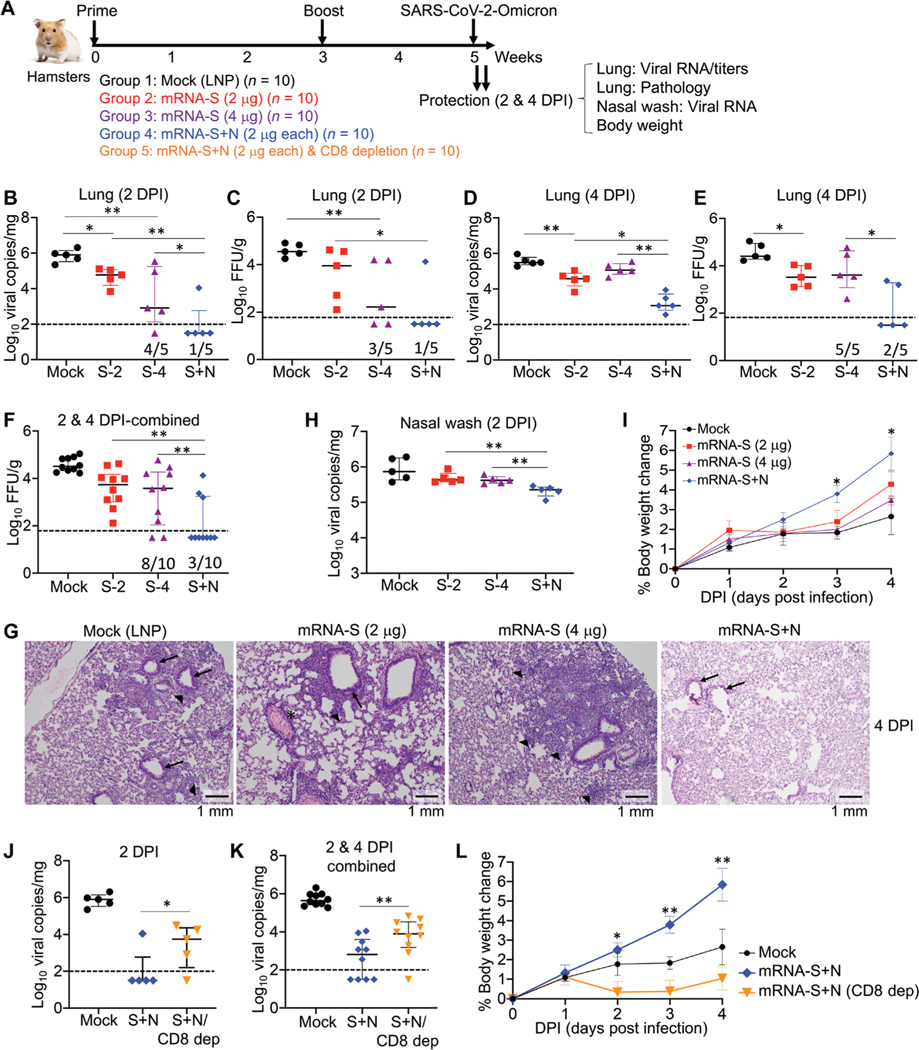
Combination mRNA-S+N vaccination confers protection against the Omicron variant in hamsters. (**A**) Hamster experimental design and timeline. Four groups of hamsters (*n* = 10 per group) were vaccinated intramuscularly with mock (empty LNP), mRNA-S (2 μg), mRNA-S (2 μg), or mRNA-S+N (2 μg for each) at weeks 0 and 3, followed by intranasal challenge with SARS-CoV-2 Omicron strain (2 × 104 pfu) at week 5. On 2 (*n* = 6) and 4 DPI (*n* = 6), lung tissues were harvested for analysis of viral RNA copies, viral titers, and pathology; nasal washes were collected for analysis of viral RNA copies; hamster body weights were monitored. In addition, a fifth group (*n* = 10) that was vaccinated with the same mRNA-S+N but received two doses of anti-CD8β–depleting antibody (intraperitoneally) before viral challenge (days −6 and −3) was included. (**B** to **E**) Viral RNA copies (log_10_ viral copies per milligram) (B and D) and viral titers (log_10_ FFU per gram) (C and E) were measured in the hamster lungs collected from the indicated groups at 2 DPI (B and C) and 4 DPI (D and E). (**F**) Pooled analysis of viral titers is shown for the hamster lung samples collected at 2 and 4 DPI. Log_10_ FFU per gram was compared between the different groups. (**G**) Hamster lung histopathology is shown using samples collected at 4 DPI. Mock, lung demonstrates bronchi with bronchiolitis (arrows) and adjacent marked interstitial pneumonia (arrowheads); mRNA-S (2 μg), lung demonstrates peribronchiolitis (arrow), perivasculitis (asterisk), and multifocal interstitial pneumonia (arrowhead); mRNA-S (4 μg), lung demonstrates marked interstitial pneumonia (arrowheads); mRNA-S+N, lung demonstrates normal bronchial, bronchiolar (arrows), and alveolar architecture. Scale bars, 1 mm. (**H**) A comparison of viral RNA copies in the nasal washes (log_10_ viral copies per milligram) between different groups on 2 and 4 DPI is shown. (**I**) A comparison of hamster body weight changes among the indicated groups is shown. * denotes difference between mRNA-S+N and mRNA-S (4 μg). (**J**) Shown is a comparison of viral RNA copies in the hamster lungs (log_10_ viral copies per milligram) on 2 DPI between mock, mRNA-S+N (S+N), and mRNA-S+N/CD8+ T cell depletion (S+N/CD8 Dep) groups. (**K**) Pooled analysis is shown for viral RNA copies in the hamster lungs for 2 and 4 DPI samples together. (**L**) Shown is a comparison of hamster body weight changes between the indicated groups. In (L), * and ** denote comparison of mRNA-S+N with mRNA-S+N/CD8 Dep. Dashed lines show limit of detection. The numbers at the bottom of (B to F) indicate the proportion of animals with a result above the limit of detection. Data are presented as median and IQR where appropriate. Kruskal-Wallis test was used for statistical analysis. *P < 0.05 and **P < 0.01.

We next investigated the efficacy of mRNA-S+N vaccination against the SARS-CoV-2 Omicron variant (BA.1). First, four groups of hamsters (*n* = 10) were vaccinated with empty LNP (mock), mRNA-S (2 μg), mRNA-S (4 μg), or mRNA-S+N (2 μg for each mRNA) at weeks 0 and 3 (Fig. 4A). The mRNA-S (4 μg) dose group was included as another control to determine whether enhanced protection by mRNA-S+N was due to the higher total dose of mRNA or LNP. Two weeks after the booster (week 5), all hamsters were intra- nasally challenged with the SARS-CoV-2 Omicron strain (2 × 10^4^ pfu). On 2 (*n* = 5) and 4 DPI (*n* = 5), protection was analyzed on the basis of viral loads, histopathologic changes in the lungs, and body weight changes (Fig. 4A). On 2 DPI, compared to the empty LNP control, mRNA-S (2 μg) induced only modest control of the Omicron variant in the lungs based on viral RNA copies (12-fold reduction) and infectious titers (3-fold reduction) (Fig. 4, B and C). Compared to 2 μg of mRNA-S, vaccination with 4 μg of mRNA-S induced comparable viral control on both 2 and 4 DPI, based on viral RNA copies (Fig. 4, B and D) and infectious titers (Fig. 4, C and E). These data indicate that a higher mRNA-S dose did not provide markedly stronger protection against the Omicron. Critically, compared to mRNA-S alone (either 2 or 4 μg), combination mRNA-S+N induced more robust control of Omicron based on viral RNA copies (Fig. 4, B and D) and infectious titers (Fig. 4, C and E). On 2 DPI, mRNA-S+N induced complete viral control with no detectable viral RNA in four of five hamsters (Fig. 4B). Viral titers yielded comparable results: There was no detectable infectious virus in four of five hamsters in the mRNA-S+N group, whereas four of five hamsters in the mRNA-S (4 μg) group had detectable virus (Fig. 4C). A similar result was observed on 4 DPI. Relative to mRNA-S alone, mRNA-S+N vaccination further reduced median viral RNA copies by 100-fold (Fig. 4D). Last, pooled analysis of viral titers for 2 and 4 DPI lung samples revealed a significant difference between mRNA-S and mRNA-S+N groups (*P* < 0.01; Fig. 4F).

Histopathological analysis showed no changes in the lungs in any hamsters on 2 DPI, including the mock-vaccinated controls (fig. S4). However, considerable changes, including bronchitis and interstitial pneumonia, became evident on 4 DPI (Fig. 4G). Compared to the mock-vaccinated controls, hamsters vaccinated with mRNA-S alone, either at the 2 or 4 μg dose, still developed lesions, including interstitial pneumonia and peribronchitis; critically, mRNA-S+N vaccination fully protected hamsters from all lesions with normal bronchial, bronchiolar, and alveolar architecture (Fig. 4G). This finding is consistent with the strong protection indicated by the above viral load data.

Analysis of viral RNA in the nasal washes indicated that mRNA-S vaccination (2 or 4 μg) had limited effects on SARS-CoV-2 Omicron shedding in the upper respiratory tract and only weakly reduced viral copies compared to the LNP controls (Fig. 4H). This likely indicates a strong immune escape of Omicron from mRNA-S–induced neutralization (9–11, 41). This weak protective effect in the nasal washes was further diminished by 4 DPI (fig. S5). However, on 2 DPI, compared to mRNA-S alone, mRNA-S+N vaccination induced a significant reduction in viral RNA copies in the nasal washes (3.3-fold reduction; *P* < 0.01; Fig. 4H), indicating that mRNA-S+N vaccination also provides additional control of Omicron in the upper respiratory tract.

Body weight analysis highlighted differences between Delta and Omicron infections in the hamsters (using the same challenge doses). Infection with the Delta variant produced a progressive decline in body weights (Fig. 3I), whereas hamsters infected with Omicron maintained a steady increase in weights through 4 DPI (Fig. 4I), consistent with reports that Omicron infection is more attenuated in animal models (42–44). Compared to mRNA-S alone (2 and 4 μg), mRNA-S+N vaccination led to a significant increase in weights (*P* < 0.05 for 3 and 4 DPI; Fig. 4I). Body weight increases for the mRNA-S+N–vaccinated hamsters were comparable to those of uninfected hamsters reported previously (about 6% increase by 4 DPI) (40). This indicates that mRNA-S+N vaccination protects hamsters from Omicron-induced morbidity, consistent with its robust protection against viral loads and pathologic effects in the lungs.

To explore the potential involvement of CD8^+^ T cells in mRNA-S+N–induced protection, we included another group of hamsters (*n* = 10) that received the same mRNA-S+N vaccine at weeks 0 and 3. Six and 3 days before the Omicron challenge, hamsters were injected (intraperitoneally) with two doses of antibody for in vivo CD8^+^ T cell depletion (Fig. 4A) (35, 36). CD8^+^ T cell depletion efficiency was confirmed by flow cytometry (fig. S6). Compared to mRNA-S+N vaccination without depletion, CD8^+^ T cell depletion resulted in a modest but significant increase in viral copies in the lungs on 2 DPI (*P* < 0.05; Fig. 4J). Pooled analysis of 2 and 4 DPI samples indicated a significant effect of CD8^+^ T cell depletion on viral RNA copies in the lungs (*P* < 0.01; Fig. 4K). Body weight analysis showed that, compared to mRNA-S+N or LNP-vaccinated hamsters, CD8^+^ T cell–depleted hamsters had reduced body weight gain on both 2 and 4 DPI (Fig. 4L), also indicating the potential involvement of CD8^+^ T cells in immune protection against Omicron by mRNA-S+N vaccination.

### Combination mRNA vaccination elicits robust N- and S-specific T cell and humoral immunity

**Fig. 5.**
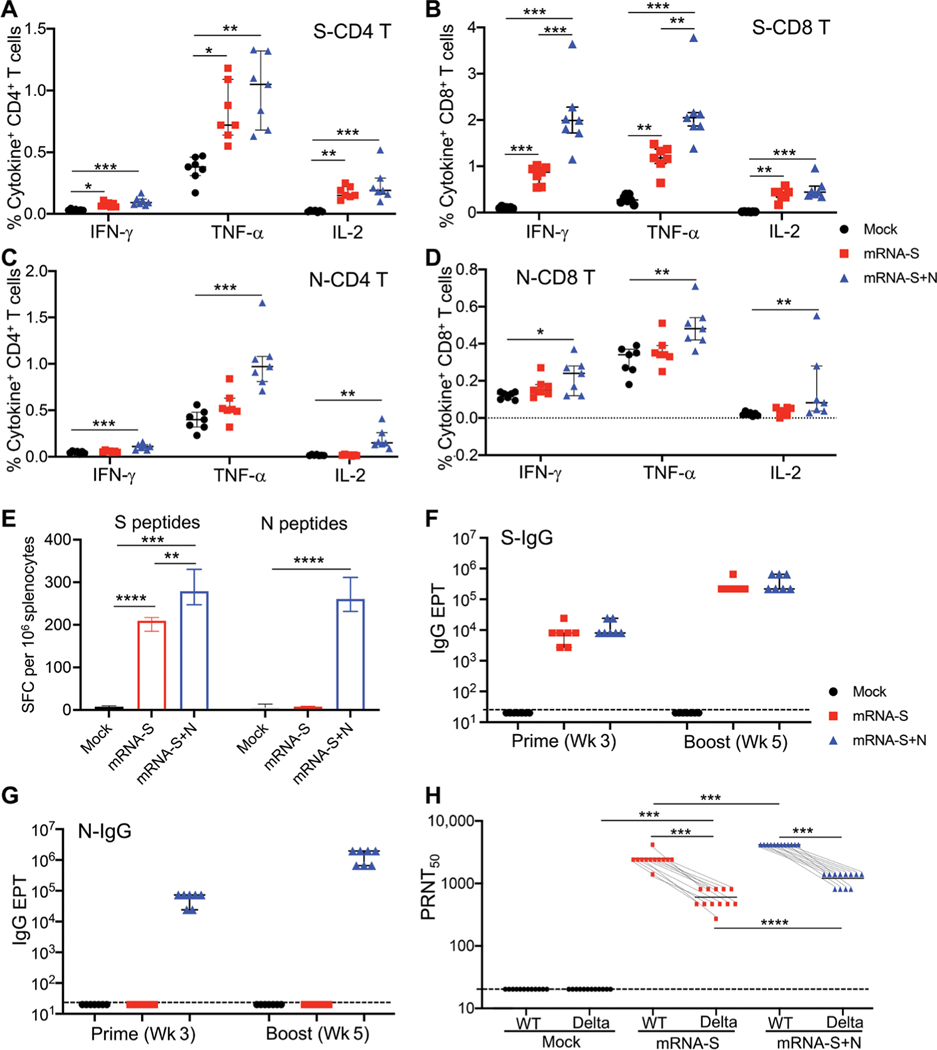
Combination mRNA-S+N vaccination induces antigen-specific immune responses in mice and hamsters. Immunogenicity experimental design and timeline: Three groups of BALB/c mice (*n* = 7 per group) were vaccinated intramuscularly with mock, mRNA-S (1 μg), or combination mRNA-S+N (1 μg for each) at weeks 0 and 3. Blood and serum samples were collected at week 3 (before booster) to measure antibody responses. Two weeks after booster (week 5), vaccine-induced T cell and antibody responses were measured. (**A** and **B**) ICS measurements of S-specific CD4+ and CD8+ T cells in the mouse spleen (week 5) are shown. Percent of individual cytokine-positive CD4+ (A) or CD8+ (B) T cells were compared between the mock and vaccine groups. (**C** and **D**) ICS measurements of N-specific CD4+ and CD8+ T cells in the mouse spleen (week 5) are shown. Percent of individual cytokine-positive CD4+ (C) or CD8+ (D) T cells were compared between the mock and vaccine groups. (**E**) IFN-γ ELISPOT measurements of antigen- specific T cells in spleen (week 5) are shown. Data were shown as SFC per 106 splenocytes. (**F** and **G**) ELISA measurement of serum S-specific (F) or N-specific (G) binding IgG are shown for samples collected after prime (week 3) or booster (week 5) vaccination in mice. Antibody EPTs were determined on the basis of serum serial dilutions and compared between different groups. (**H**) Serum samples were collected from the hamsters in Fig. 3D (*n* = 12) after booster vaccination (week 5) but before viral challenge. Samples were used to measure neutralizing activity by PRNT. PRNT_50_ neutralization titers for individual serum samples were compared among different groups and between the wild-type virus and the Delta variants. Dashed lines (F, G, and H) show limit of detection for each assay. Data are presented as median and IQR where appropriate. Kruskal-Wallis test was used for statistical analysis. *P < 0.05, **P < 0.01, ***P < 0.001, and ****P < 0.0001.

To better understand antigen-specific immune responses induced by vaccination, a mouse immunogenicity experiment was conducted, where three groups of BALB/c mice (*n* = 7 per group) were vaccinated with PBS (mock), mRNA-S alone, or mRNA-S+N at weeks 0 and 3 using a similar experimental design to that described in Fig. 1A. Mouse splenocytes collected at week 5 were stimulated with S or N peptide pools, and ICS was performed to identify S- and N-specific T cells (Fig. 5, A to D). The data showed that combination mRNA-S+N vaccination elicited robust S-specific (Fig. 5, A and B) and N-specific (Fig. 5, C and D) CD4^+^ and CD8^+^ T cell responses. Among the cytokines examined, TNF-α was highly expressed by both S- and N-specific T cells, followed by IFN-γ and IL-2 (Fig. 5, A to D). Compared to mRNA-S alone, the mRNA-S+N vaccination appeared to augment the S-specific CD8^+^ T cell response (*P* < 0.001 for IFN-γ^+^ and *P* < 0.01 for TNF-α^+^) (Fig. 5B). Induction of S- and N-specific T cells by mRNA-S+N vaccination compared to mRNA-S alone was also confirmed by IFN-γ ELISPOT (Fig. 5E and fig. S7).

Serum binding IgG to S or N was measured by ELISA, and the data revealed similar patterns (Fig. 5, F and G). The mRNA-S alone induced robust binding IgG targeted to S (Fig. 5F), but not to N (Fig. 5G), after prime vaccination, which was markedly enhanced by the booster vaccination (Fig. 5F). Compared to mRNA-S alone, combination mRNA-S+N elicited strong binding IgG specific to both S and N proteins after prime vaccination, both of which were also enhanced by the booster vaccination (Fig. 5, F and G).

We next evaluated vaccine-induced serum neutralizing activities. In the hamster study described earlier (Fig. 3D), serum samples were collected after booster vaccination (week 5) and before viral challenge. Their neutralizing activities against wild-type SARS-CoV-2 (WA1/2020) and the Delta variant were measured by PRNT (45). Serum from the mRNA-S–vaccinated hamsters manifested strong neutralizing activity against the wild-type virus [wild-type half maximal PRNT values (PRNT_50_), 2667] but markedly reduced neutralizing activity against the Delta variant (Delta PRNT_50_, 440; a 5.1-fold reduction; Fig. 5H). Compared to mRNA-S alone, combination mRNA-S+N elicited stronger serum neutralizing activity against both the wild-type virus (*P* < 0.001) and the Delta variant (*P* < 0.0001; Fig. 5H). These data are consistent with the augmented S-specific CD8^+^ T cell response (Fig. 5B) induced by combination mRNA-S+N compared to mRNA-S alone. Together, the immune analyses suggest that combination mRNA-S+N vaccination not only induces N-specific immunity but also elicits stronger S-specific CD8^+^ T cell response and serum neutralizing antibody activities when compared to mRNA-S alone. These responses may collectively contribute to the enhanced protection against the SARS-CoV-2 Delta and Omicron variants.

## DISCUSSION

Here, we report a nucleoside-modified mRNA vaccine that expresses the SARS-CoV-2 N (mRNA-N) and its immunogenicity and efficacy when used alone or in combination with the S-expressing mRNA vaccine (mRNA-S). We demonstrated that mRNA-N is highly immunogenic and, by itself, induced modest control of SARS-CoV-2 infection. The combination mRNA-S+N vaccination induces more robust control of the SARS-CoV-2 Delta and Omicron variants in the lungs than mRNA-S alone and also provides additional protection against both variants that results in reduced viral load in the upper respiratory tract. In vivo cell depletion suggested potential involvement of CD8^+^ T cells in mRNA-S+N vaccine–induced protection. Antigen-specific immune analysis indicated that induction of N-specific immunity, together with the augmented S-specific immunity, is associated with the enhanced protection by the bivalent mRNA vaccination. Our study thus suggests an mRNA vaccine approach for improved control of SARS-CoV-2 VOCs.

The emergence of SARS-CoV-2 VOCs has posed challenges to the current S-targeting vaccines (4–8), and next-generation vaccine strategies may benefit from multivalency (46). Several studies have tested multivalent SARS-CoV-2 candidate vaccines that include the N protein, including adenoviral (23, 47) and modified vaccinia Ankara (48, 49) vectors. These studies did not compare efficacy with the current, clinically proven S-targeting vaccines. In addition, most of these vaccine approaches were only tested against earlier circulating VOCs, and their efficacy against the predominant Omicron remains unclear.

We generated an mRNA-LNP vaccine that encodes the N protein alone and demonstrated that mRNA-N is immunogenic and elicits strong T cell and binding antibody responses without neutralizing activity. Our vaccine thus provides an opportunity to explore whether the immune response to the N protein could confer protection against SARS-CoV-2 VOCs independent of neutralizing antibodies. The data indeed show that mRNA-N induces modest protection against MA-SARS-CoV-2 and Delta strains. Critically, our study also shows that combined S and N immunization using the mRNA-LNP platform elicits markedly more robust protection against both Delta and Omicron VOCs. Given that the mRNA-LNP platform has been clinically proven with a good safety profile in large human populations, our approach could be rapidly adapted to clinical testing against emerging and reemerging VOCs.

To develop vaccines against VOCs, utilization of VOC-specific S immunogens represents another potential strategy (50) and has been tested in animal models (51). It was shown that an Omicron S-specific mRNA booster offered no improved protection against Omicron challenge when compared to boosting with the ancestral S mRNA vaccine (51), indicating that this VOC-targeted booster strategy is likely challenging. In addition, in the face of constantly mutating S proteins, design and selection of VOC-specific sequences are too slow to be ideal. In our study, both mRNA-N and mRNA-S were designed on the basis of the ancestral SARS-CoV-2 sequence (Wuhan-Hu-1). Despite the use of this early sequence, our data showed that the inclusion of mRNA-N along with mRNA-S for immunization elicited robust protection against both Delta and Omicron. The current study did not compare the combination mRNA approach with a third-dose ancestral S mRNA booster, which will be investigated in the future to further validate our approach.

Other than neutralizing antibodies, the importance of T cell immunity in protection against ancestral and VOC strains has been recognized (52). CD8^+^ T cell depletion in convalescent nonhuman primates (NHPs) led to partially abrogated protection against SARS-CoV-2 rechallenge (53). Multiple studies have also shown that ancestral SARS-CoV-2 S–specific T cells induced by prior infection or vaccination cross-recognize the Omicron S protein (54, 55). Consistent with these findings, our study provides multiple lines of evidence suggesting the involvement of T cells in mRNA-S+N vaccine–induced protection against variants: (i) mRNA-N alone induced modest protection against both MA-SARS-CoV-2 and Delta strains in the absence of neutralizing antibodies and (ii) in vivo CD8^+^ T cell depletion supported a role for CD8^+^ T cells in viral control and protection against body weight loss after Omicron challenge. Although our data support the involvement of CD8^+^ T cells in mRNA-S+N vaccination, the relative contributions of N-or S-specific T cells in this process are unclear and need to be explored. In addition, because validated antibodies for hamster T cells are limited, the in vivo CD8 depletion experiment should be validated in other animal models (such as NHPs), where well-characterized antibodies are more readily available. Further testing of our vaccine approach in NHPs would also provide additional opportunities to evaluate its safety and efficacy in models that are more closely related to humans.

Viral loads in the upper respiratory tract and induction of mucosal immunity are key factors affecting SARS-CoV-2 transmission and breakthrough infections in vaccinated people. In our hamster models of VOC challenge, whereas the combined mRNA-S+N vaccine induced robust viral control in the lungs, its additive antiviral effect appeared to be less profound in the upper respiratory tract. Mechanisms of upper airway protection remain unclear but may be associated with respiratory mucosal immunity induced by vaccination. Most first-generation COVID-19 vaccines, including mRNA-LNP vaccines, were designed for intramuscular delivery and induced strong systemic immune responses. Whether systemic COVID-19 vaccination effectively induces respiratory immunity is less well defined (46). A recent study indicated that, although intramuscular mRNA-S immunization elicited strong circulating antibody and T cell responses, respiratory neutralizing antibodies in the vaccinated individuals appeared to be weaker than those of COVID-19 convalescent patients (56). Animal studies demonstrated that a mucosal booster with a viral vector after the mRNA-S prime augmented neutralizing antibodies in the respiratory system (56). Thus, heterologous approaches involving different vaccine platforms (such as mRNA and viral vectors) and a combination of different immunization routes (such as intramuscular and intranasal or oral) are worthy of further investigation to improve airway infection and transmission protection against VOCs.

Another interesting finding of our study is that, compared to mRNA-S alone, combination mRNA-S+N vaccination led to augmented S-specific immunity. This was unexpected because the mRNA-S doses in the two vaccines were identical. A study evaluating various doses of mRNA-1273 vaccine in hamsters showed comparable neutralizing antibodies induced by 1- and 5-μg doses (34). These results argue against the additional LNP in the vaccine being responsible for the augmented S-specific immunity by the combined mRNA S+N vaccine. Explanations of this finding are not yet clear. One hypothesis is that cross-priming effects occur between N and S antigens after vaccination or that mRNA-N coimmunization may induce an immune environment that favors the generation of S-specific immunity. A better understanding of mechanisms for S- and N-specific immunity regulation after combined mRNA-S+N vaccination is needed. For example, a detailed dissection of early events after vaccination, such as protein expression, antigen presentation, and stimulation of the innate and inflammatory response, would help address the question.

Our study has several limitations. First, vaccine efficacy was only examined in animal models at 2 weeks after booster immunization. Thus, the durability of protection against SARS-CoV-2 VOCs is uncertain. Analysis of the durability of the immune response induced by mRNA-S+N as compared to mRNA-S alone and evaluation of vaccine efficacy against VOCs at longer intervals after booster immunization would help address this critical question. Second, our study evaluated vaccine efficacy in immunologically naïve animals.

Given that large segments of the human population have been vaccinated with the first-generation vaccines or infected with SARS-CoV-2, it is important to also assess our combined vaccine approach as boosters in animal models or humans with preexisting immunity. Last, our data show that mRNA-N alone elicits a strong antibody response. Although we measured binding and neutralization activities of these antibodies, other effector functions of antibodies elicited by vaccination are not clear and warrant further investigation. Despite these limitations, our study presents a proof of concept that a vaccine approach targeting both the highly variable S and the more conserved N can induce stronger and broader protection against SARS-CoV-2 VOCs. This approach may have broad utility against future emerging VOCs and thus should be further developed.

## MATERIALS AND METHODS

### Study design

We designed and generated an mRNA-LNP vaccine that encodes the ancestral SARS-CoV-2 viral N (mRNA-N). The objective of this study was to evaluate the immunogenicity and efficacy of the mRNA-N vaccine alone or in combination with the clinically proven mRNA-S vaccine against SARS-CoV-2 VOCs. Expression of target viral protein by the vaccine was confirmed in 293T cells before testing in animal experiments. In vitro assays (PCR and ELISA) included duplicates or were repeated at least twice in the laboratory. The vaccines were evaluated in two animal models (mice and hamsters) for protection against three SARS-CoV-2 strains (MA-SARS-CoV-2, Delta, and Omicron). The animal study protocols were approved by the Institutional Animal Care and Use Committee at the University of Texas Medical Branch (UTMB; protocol numbers, 1703020A and 2009087). Animal experiments were performed in accordance with the recommendations in the Guide for the Care and Use of Laboratory Animals of the National Institutes of Health. Sample size for each animal study (*n* ≥ 5 per group) was not calculated by power analysis. Instead, it was determined on the basis of previous virus challenge experiments in our laboratories and those reported in the literature. Animals were randomly assigned to each group, and the study design was not blinded to researchers and animal facility staff.

### mRNA synthesis and LNP formulation

Antigens encoded by the mRNA vaccines were derived from the ancestral SARS-CoV-2 Wuhan-Hu-1 strain (GenBank MN908947.3). Nucleoside-modified mRNAs expressing SARS-CoV-2 full-length N (mRNA-N) or prefusion-stabilized S protein with two proline mutations (mRNA-S-2P) were synthesized by in vitro transcription using T7 RNA polymerase (MegaScript, Thermo Fisher Scientific) on linearized plasmid templates, as previously reported (26). Uridine triphosphate was replaced with one-methylpseudouridine (m1Ψ)-5′-triphosphate (TriLink, catalog no. N-1081) for producing nucleoside-modified mRNAs. Polyadenylated tails were added to the end of modified mRNAs for optimized protein expression. In vitro transcribed mRNAs were capped using ScriptCap m7G capping system and ScriptCap 2′-*O*-methyltransferase kit (ScriptCap, CellScript) (26), followed by purification using the cellulose purification method as previously described (27). Purified mRNAs were analyzed by agarose gel electrophoresis and were kept frozen at −20°C. The mRNAs were formulated into LNPs using an ethanolic lipid mixture of ionizable cationic lipid and an aqueous buffer system, as previously reported (28, 57). Formulated mRNA-LNPs were prepared according to RNA concentrations (1 μg/μl) and were stored at −80°C for animal immunizations.

### Confirmation of protein expression by mRNA-N

293T cells [American Type Culture Collection (ATCC), CRL-3216] in six-well plates were directly transfected with 2 μg of mRNA-N-LNP or not transfected (as a cell-only control). Eighteen hours after transfection, cells were lysed in radioimmunoprecipitation assay buffer (Thermo Fisher Scientific) for Western blot analysis. Cell lysates were centrifuged, followed by collection of supernatants for quantification of total protein concentration using Microplate bicinchoninic acid protein assay kit (Pierce, Thermo Fisher Scientific). Equal amounts of protein were separated by SDS–polyacrylamide gel electrophoresis using 4 to 15% SDS polyacrylamide gels (Bio-Rad). Proteins were transferred onto a nitrocellulose membrane (Bio-Rad). The membrane was blocked in tris-buffered saline (TBS) containing 0.05% Tween-20 (TBST; Thermo Fisher Scientific) and 5% (w/v) nonfat dried milk (Bio-Rad) for 1 hour at room temperature, followed by incubation with anti–SARS-CoV-2 nucleocapsid mouse monoclonal antibody (MA5–29981, Thermo Fisher Scientific; 1:1000) overnight at 4°C. After washing in TBST (three times for 5 min), the membrane was incubated for 1 hour with horseradish peroxidase (HRP)–linked anti-mouse IgG (1:5000; 7076S, Cell Signaling). The membrane was washed, and proteins were visualized using the enhanced chemiluminescence Western blotting substrate (Thermo Fisher Scientific).

### Mouse immunization and SARS-CoV-2 challenge

Vaccine immunogenicity and efficacy were evaluated in 6-week-old female BALB/c mice (the Jackson Laboratory; strain no. 000651). For immunogenicity, four groups of mice (seven per group) were immunized intramuscularly with either PBS (mock control), mRNA-S (1 μg), mRNA-N (1 μg), or combined mRNA-S+N (1 μg for each) at week 0 (prime) and week 3 (boost), respectively. The vaccine or control PBS was administered at 50 μl per injection. Blood and serum samples were collected 3 weeks after prime vaccination (before booster vaccination) to measure vaccine-induced antibody response. All mice were euthanized 2 weeks after booster vaccination (week 5). Blood and serum and spleen samples were collected for analyses of vaccine-induced humoral and cellular immune responses.

For challenge studies, another four groups of BALB/c mice (eight per group) received the same mock control or vaccines as indicated above. Vaccine doses and immunization timeline were identical to the above immunogenicity study. Two weeks after booster vaccination (week 5), all mice were transferred to animal biosafety level 3 (ABSL-3) facility and were intranasally challenged with an MA-SARS-CoV-2 CMA4 strain (2 × 10^4^ pfu), as previously reported (32, 58). Two days after viral challenge, all mice were euthanized and equivalent portions of the lung tissues were collected for quantification of SARS-CoV-2 viral loads.

### Hamster immunization and SARS-CoV-2 Delta or Omicron challenge

Vaccine-induced protection against Delta or Omicron strain was evaluated in hamsters. For Delta challenge, four groups of 4- to 5-week-old male golden Syrian hamsters (12 per group), strain HsdHan: AURA (Envigo, catalog no. 8901M), were vaccinated intramuscularly with either PBS (mock control), mRNA-S (2 μg), mRNA-N (2 μg), or combined mRNA-S+N (2 μg for each) at weeks 0 and 3, respectively. For Omicron variant challenge, five groups of 4- to 5-week-old male golden Syrian hamsters (10 per group) were intramuscularly vaccinated with empty LNP (mock control) mRNA-S (2 μg), mRNA-S (4 μg), mRNA-S+N (2 μg for each), or mRNA-S+N (2 μg for each) with CD8^+^ T cell depletion. For the mRNA-S+N CD8^+^ T cell–depleted group, 6 days (day −6) and 3 days (day −3) before viral challenge, hamsters were intraperitoneally injected with 175 μg of anti-rat CD8β antibody (16–0080-38; eBio341; functional grade; Thermo Fisher Scientific) for in vivo CD8^+^ T cell depletion as reported previously (35, 36). CD8^+^ T cell depletion in hamster was confirmed by splenocyte immune staining [anti–CD8β-phycoerythrin (PE); 12–0080-82; eBio341; Thermo Fisher Scientific] and flow cytometric analysis (fig. S6). The vaccine or mock control was administered at 100 μl per injection. Serum samples were collected from all hamsters before viral challenge to measure vaccine-induced neutralizing antibodies. Two weeks after booster vaccination (week 5), hamsters were transferred to the ABSL-3 facility and intranasally challenged with the SARS-CoV-2 Delta (2 × 10^4^ pfu) or Omicron strain (2 × 10^4^ pfu; World Reference Center for Emerging Viruses and Arboviruses). On 2 DPI, six hamsters challenged with Delta or five challenged with Omicron were euthanized. Nasal wash samples and equivalent portions of the lung tissues were collected for various analyses of vaccine-induced protection. On 4 DPI, the same procedures were repeated for the half of hamsters in each group (six for Delta and five for Omicron). Hamster body weights were monitored daily to evaluate vaccine-induced protection from body weight loss.

### Binding IgG by ELISA

Vaccine-induced, N- and S-specific binding IgG in serum samples was measured by ELISA. Plates (Greiner Bio-One) were coated with recombinant S (1 μg/ml; 40589-V08B1, Sino Biological) or N protein (40588-V08B, Sino Biological) overnight at 4°C. Plates were washed three times (5 min each time) and then blocked with blocking buffer [8% fetal bovine serum (FBS) in Dulbecco’s PBS (DPBS)] for 1.5 hour at 37°C, followed by washing and incubation at 37°C for 1 hour with serially diluted serum samples (initial dilution, 1:100; 1:3 serial dilution) in blocking buffer at 50 μl per well. Plates were washed again and incubated with HRP-conjugated anti-mouse IgG secondary antibody (405306; BioLegend; 1:3000) for 1 hour at 37°C. After final wash, plates were developed using TMB 1-Component Peroxidase Substrate (Thermo Fisher Scientific), followed by termination of reaction using the TMB stop solution (Thermo Fisher Scientific). Plates were read at 450 nm wavelength within 15 min by using a Microplate Reader (BioTek). Binding IgG EPTs for each sample were calculated.

### Neutralizing assay

Serum neutralizing activity was examined by a standard PRNT, as previously reported (59, 60). The assays were performed with Vero E6 cells (ATCC, CRL-1586) using the SARS-CoV-2 wild-type or Delta strains. Briefly, serum samples were heat-inactivated and twofold serially diluted (initial dilution, 1:10), followed by incubation with 100 pfu of wild-type SARS-CoV-2 (USA-WA1/2020) or the Delta strain for 1 hour at 37°C. The serum-virus mixtures were placed onto Vero E6 cell monolayer in six-well plates for incubation for 1 hour at 37°C, followed by addition of 2-ml overlay consisting of minimum essential medium (MEM) with 1.6% agarose, 2% FBS, and 1% penicillin-streptomycin to the cell monolayer. Cells were then incubated for 48 hours at 37°C, followed by staining with 0.03% liquid neutral red for 3 to 6 hours. Plaque numbers were counted, and PRNT_50_ were calculated. Each serum sample was tested in duplicates.

### ICS and flow cytometry

Mouse splenocytes were washed with fluorescence-activated cell sorting (FACS) buffer (1% FBS and 0.5 M EDTA in PBS) and resuspended in complete RPMI 1640 with 10 mM Hepes supplemented with 10% FBS, 2-mercaptoethanol, sodium pyruvate, non-essential amino acids, penicillin-streptomycin, and L-glutamine. Cells were stimulated with S peptide pool (1 μg/ml; JPT, PM-WCPV-S; Swiss-Prot ID, P0DTC2) or N peptide pool (Miltenyi, 130–126-698; Protein QHD43423.2) in the presence of anti-CD28 (1 μg/ml; Invitrogen, 14–0281-86) for costimulation for 6 hours. In the last 4 hours of incubation, protein transport inhibitor brefeldin-A was added. Cells stimulated with phorbol 12-myristate 13-acetate and ionomycin or dimethyl sulfoxide only were included as positive control and negative control, respectively. After stimulation, cells were first stained for surface markers, including CD4-peridinin-chlorophyll-protein–Cy5.5 (BioLegend, 100540; clone, RM4–5; 0.2 mg/ml), CD8-brillaint violet (BV)711 (BioLegend, 100759; clone, 53–6.7; 0.2 mg/ml), and CD44-BV510 (BioLegend, 103044; clone, IM7; 0.2 mg/ml). The surface staining was performed on ice for 30 min. After washing with PBS, cells were resuspended with Zombie-dye (BioLegend) for viability staining and incubated at room temperature for 15 min. After surface and viability staining, cells were fixed with fixation buffer (BioLegend, 420801) and permeabilized with perm/wash buffer (BioLegend, 421002), followed by ICS with IFN-γ–BV605 (BioLegend, 505840; clone, XMG1.2; 0.2 mg/ml), TNF-α–PE-Cy7 (BioLegend, 506324; clone, MP6-XT22; 0.2 mg/ml), and IL-2–allophycocyanin (Tonbo Biosciences, 20–7021; clone, JES6–5H4; 0.2 mg/ml) on ice for 30 min. Cells were then washed with perm/wash buffer and were processed with a multiparametric flow cytometer FACS LSRFortessa (BD Biosciences). Data were analyzed using FlowJo (TreeStar).

### IFN-γ ELISPOT

ELISPOT was performed according to the manufacturer’s instructions (Cellular Technology Ltd.; MU IFN-γ). Plates were coated with anti–IFN-γ capture antibody (Cellular Technology Ltd.) at 4°C overnight. Splenocytes (0.25 × 10^6^) were stimulated in duplicates with SARS-CoV-2 S-peptide (2 μg/ml; Miltenyi Biotec, 130–126-701) or N-peptide pools (2 μg/ml; Miltenyi Biotec, 130–126-699) for 24 hours at 37°C. Splenocytes stimulated with anti-CD3 (1 μg/ml; Thermo Fisher Scientific, 16–0031-82) or medium alone were used as positive and negative control, respectively. This was followed by incubation with biotin-conjugated anti–IFN-γ (Cellular Technology Ltd.) for 2 hours at room temperature and then alkaline phosphatase–conjugated streptavidin for 30 min. The plates were washed and scanned using an ImmunoSpot 4.0 analyzer, and the spots were counted with ImmunoSpot software (Cellular Technology Ltd.) to determine SFC per 10^6^ splenocytes.

### RNA extraction and RT-PCR quantification of viral RNA copies

RNA was extracted from the lung tissues (mice and hamsters) and nasal washes (hamsters) using the TRIzol LS Reagent (Thermo Fisher Scientific) according to the manufacturer’s instructions. Concentration and purity of the extracted RNAs were determined using the multimode plate reader (BioTek). To quantify SARS-CoV-2 viral RNA copies, one-step RT-PCR was performed using the iTaq Universal SYBR Green One-Step Kit (Bio-Rad) and the CFX Connect Real-Time PCR Detection System (Bio-Rad). Primer sets for the SARS-CoV-2 *E* gene (forward, 5′-GGAAGAGACAGGTACGTTAATA-3′; reverse, 5′-AGCAGTACGCACACAATCGAA-3′) were used. PCR reactions (20 μl) contained primers (10 μM), RNA sample (2 μl), iTaq universal SYBR Green reaction mix (2×; 10 μl), iScript reverse transcriptase (0.25 μl), and molecular grade water. PCR cycling conditions were as follows: 95°C for 3 min, 45 cycles of 95°C for 5 s, and 60°C for 30 s. For each RT-PCR, a standard curve was included using an RNA standard (in vitro transcribed, 3839 base pairs containing genomic nucleotide positions 26,044 to 29,883 of SARS-CoV-2 genome) to quantify the absolute copies of viral RNA in the lung tissue or nasal wash.

### Plaque assay

Homogenized lung tissues were serially diluted in Dulbecco’s modified Eagle’s medium (DMEM; Gibco) with 1% antibiotic-antimycotic (Gibco) and allowed to infect a confluent monolayer of Vero E6 cells (ATCC; CRL-1586) in a 96-well plate for 45 min at 37°C with 5% CO_2_. After infection, cells were overlaid with a solution of 85% MEM (Gibco) and 15% DMEM supplemented with 1% antibiotic-antimycotic and 0.85% methyl cellulose (Sigma-Aldrich). After 24 to 36 hours, the monolayers were fixed with formalin (Thermo Fisher Scientific) for at least 24 hours. Monolayers were washed with DPBS (Sigma) and incubated in permeabilization buffer consisting of DPBS supplemented with 0.1% bovine serum albumin (Sigma-Aldrich) and 0.1% saponin (Sigma-Aldrich) for 30 min at room temperature. Permeabilization buffer was removed, and monolayers were incubated overnight at 4°C with rabbit polyclonal antibody against SARS-CoV N protein (a gift from S. Makino, Department of Microbiology and Immunology, UTMB) diluted in permeabilization buffer (1:3000). Excess antibody was washed away with DPBS, and monolayers were incubated for 1 hour at room temperature with HRP-conjugated goat anti-rabbit IgG (Cell Signaling Technology, 7040) diluted in permeabilization buffer (1:2000). Excess antibody was washed away with DPBS, and foci were stained using KPL TrueBlue Peroxidase Substrate (SeraCare). Once foci were visible under a light microscope, excess substrate was removed, and the monolayers were washed with water. Wells were imaged using the Cytation7 Imagining Reader (BioTek). Foci were counted manually, and results were shown as focus-forming units (FFU).

### Lung histopathology

Lungs were harvested from hamsters, fixed in 10% neutral-buffered formalin, and embedded in paraffin. Thin (5 μm) paraffin-embedded sections were placed on glass slides, and paraffin was then removed from the samples using three changes of xylene for 2 min each. Samples were hydrated, followed by staining for 3 min in hematoxylin solution. The slides were then washed under running tap water at room temperature for at least 5 min, followed by staining with an eosin Y solution for 2 min. Slides were then subjected to dehydration again and cleared with three changes of xylene for 2 min per change. Last, a drop of mounting medium was added to attach the coverslip. The slides were read by a pathologist in a blinded manner.

### Statistical analysis

Raw, individual-level data are presented in data file S1. Statistical analysis was performed using the GraphPad Prism 8.0 software. Nonparametric tests were used throughout this paper for statistical analysis. Data were presented as median and interquartile ranges (IQRs). Comparison among groups was performed by using either Mann-Whitney test (two groups) or Kruskal-Wallis test (more than two groups). Two-tailed *P* values were denoted, and *P* values <0.05 were considered as significant.

## Supplementary Material

STM-supplementary

Data File S1

mdar_checklist
