# Supplementary material for: Dual spike and nucleocapsid mRNA vaccination confer protection against SARS-CoV-2 Omicron and Delta variants in preclinical models
Source: Sci Transl Med. Author manuscript; Available in PMC 2023 Feb 14. (PMC9926941; doi:10.1126/scitranslmed.abq1945)
Supplement: STM-supplementary [file NIHMS1863403-supplement-STM-supplementary.pdf]

Science Translational Medicine
AAAS

Supplementary Materials for

Dual spike and nucleocapsid mRNA vaccination confer protection against SARS-CoV-2 Omicron and Delta variants in preclinical models

Renee L. Hajnik *et al.*

Corresponding author: Haitao Hu, haihu@utmb.edu; Kenneth S. Plante, ksplante@utmb.edu

Sci. Transl. Med. **14**, eabq1945 (2022)
DOI: 10.1126/scitranslmed.abq1945

The PDF file includes:

Figs. S1 to S7
Legend for data file S1

Other Supplementary Material for this manuscript includes the following:

Data file S1
MDAR Reproducibility Checklist

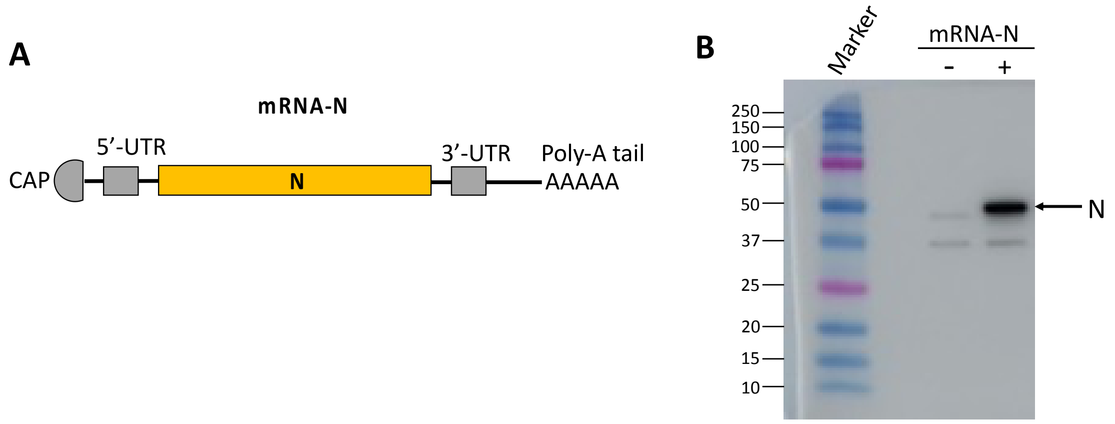

Fig. S1. mRNA-N vaccine design and characterization. (A) Structure of mRNA-N vaccine. Pseudouridine modified RNAs encoding full-length SARS-CoV-2 N protein were synthesized, followed by 5'-capping and 3' poly-A tailing. **(B)** A western blot was used to confirm SARS-CoV-2 Nucleocapsid (N) protein expression by mRNA-N. 293T cells were transfected with 2 μg mRNA-N-LNP or phosphate-buffered saline for 18 hours. Total protein was extracted from cells for western blot analysis. SARS-CoV-2 N protein was detected using a specific anti-N antibody (MA5-29981).

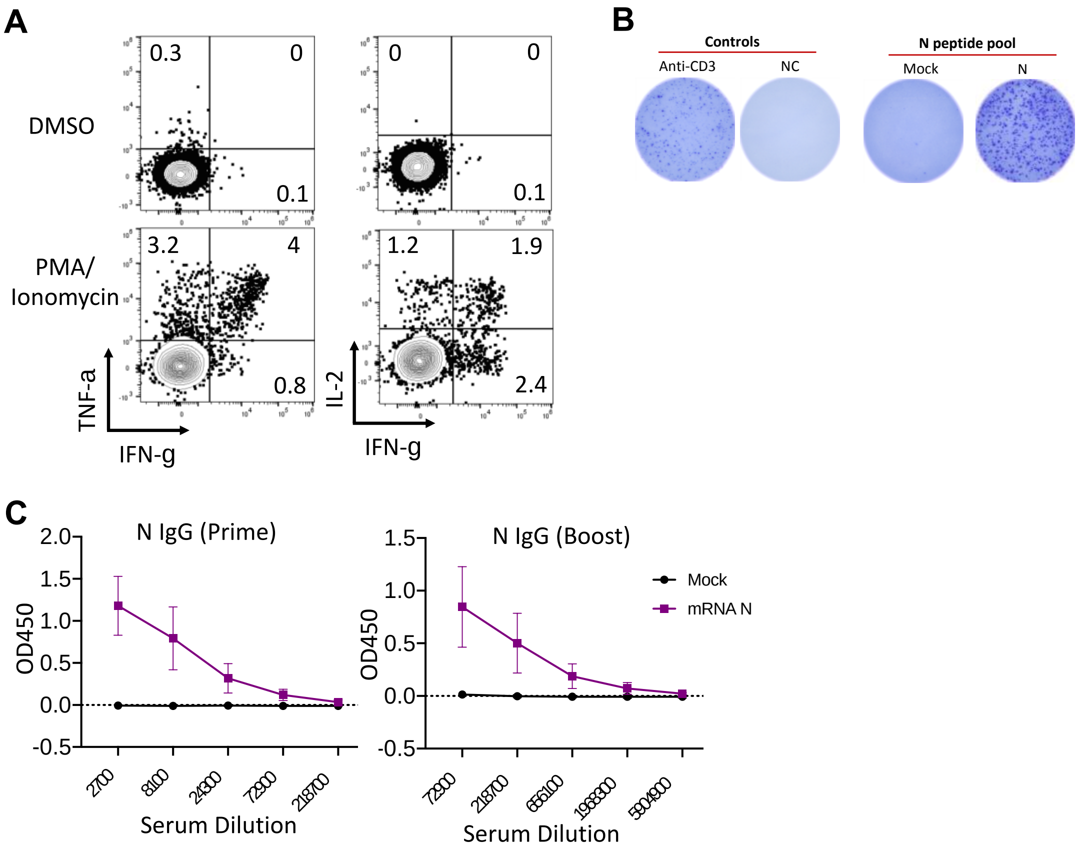

Fig. S2. mRNA-N vaccine immunogenicity in mice. (A) Representative flow cytometry plots are shown for cytokine expression in T cells following dimethyl sulfoxide (DMSO, negative control) or phorbol 12-myristate 13-acetate (PMA)/Ionomycin (positive control) stimulation. TNF-α, tumor necrosis factor-α; IFN-γ, interferon-γ; IL-2, interleukin-2. **(B)** Representative plots for detecting N-specific T cells in mouse spleen by IFN-γ enzyme-linked immunosorbent spot (ELISPOT) assay are shown. Positive control (anti-CD3 stimulation) and negative control (NC, medium only) for the ELISPOT are also shown. Mock indicates DMSO. **(C)** Enzyme-linked immunosorbent assay (ELISA) measurement of N-specific binding IgG in serially diluted (1:3) serum samples (n = 7 per group). Mean optical density (OD) values (±SD) for serum samples at indicated dilutions after prime (left) and booster (right) vaccination were shown for determination of IgG endpoint titers (EPTs).

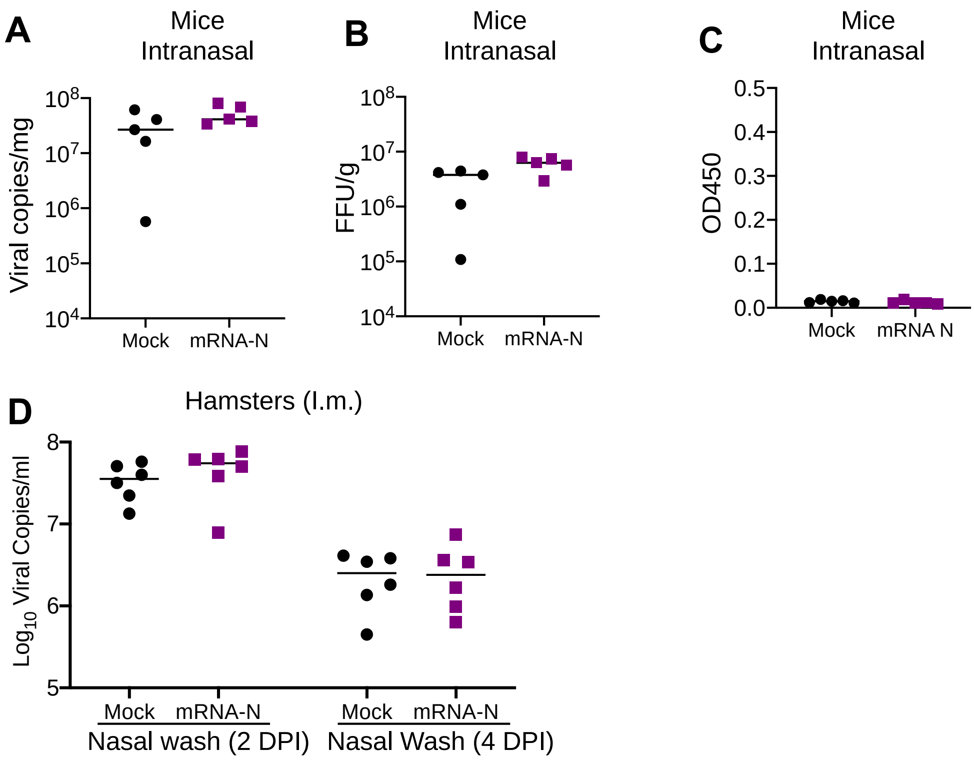

Fig. S3. Analysis of mRNA-N-induced protection in mice and hamsters. (A) Protection was analyzed in mice following intranasal (i.n.) vaccination. Two groups of BALB/c mice (n=5 per group) were i.n. immunized with PBS (mock) or mRNA-N vaccine (1 µg) at week 0 and week 3, followed by intranasal challenge with a mouse-adapted SARS-CoV-2 strain [2×10^4 plaque forming units (pfu)]. 2 days post-infection (2 DPI), viral copies (Log_{10} copies/mg) in the mouse lung were analyzed by reverse transcription polymerase chain reaction (RT-PCR) and were compared between mock and vaccine group. **(B)** A comparison of viral titers in the mouse lungs [Log_{10} focus forming units (FFU)/g] is shown between the mock and vaccine groups following i.n. vaccination. **(C)** Serum antibody responses are shown in mice following intranasal (i.n.) vaccination. Serum samples were collected from the i.n. vaccinated mice 2 weeks after booster vaccination (prior to viral challenge). Serum N-specific binding IgG was measured by ELISA. OD450 values for individual serum samples (1:30 dilution) are shown. **(D)** A comparison of viral RNA copies in the nasal wash of mock

and mRNA-N-vaccinated hamsters is shown for samples collected on 2 DPI and 4 DPI (n = 6 per group). Horizontal bars indicate mean.

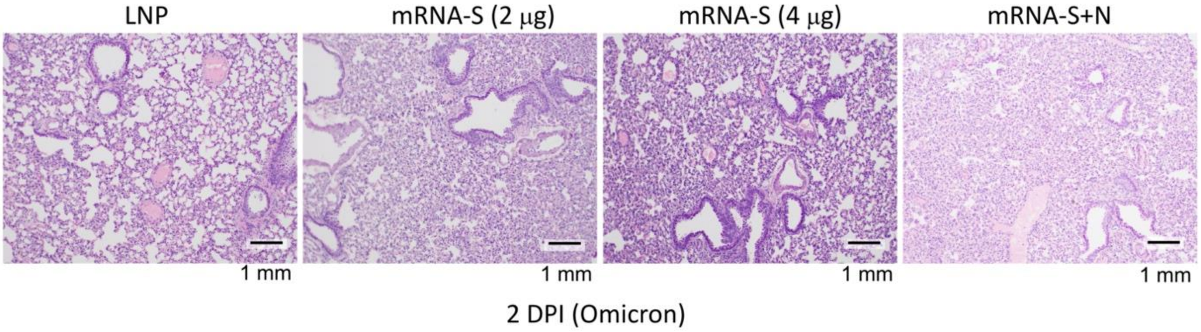

Fig. S4. Histopathological analysis of lungs collected on 2 DPI from hamsters following Omicron challenge. Lung tissues (2 DPI) were fixed and 5 μm sections cut from mock LNP, mRNA-S (2 μg), mRNA-S (4 μg), or mRNA-S+N-vaccinated hamsters and stained with hematoxylin & eosin (H&E). Lungs of hamsters from all four groups demonstrate normal bronchial, bronchiolar, and alveolar architecture. Scale bar of each image indicates 1 mm.

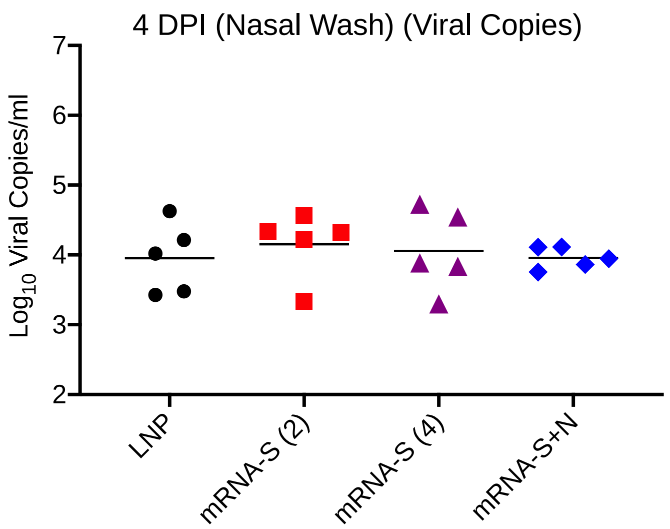

Fig. S5. Viral RNA copies (Log_{10}) are shown in the nasal washes of hamsters in different vaccination groups at 4 days after Omicron challenge. Horizontal bars indicate mean. n = 5 per group, as in Fig. 4.

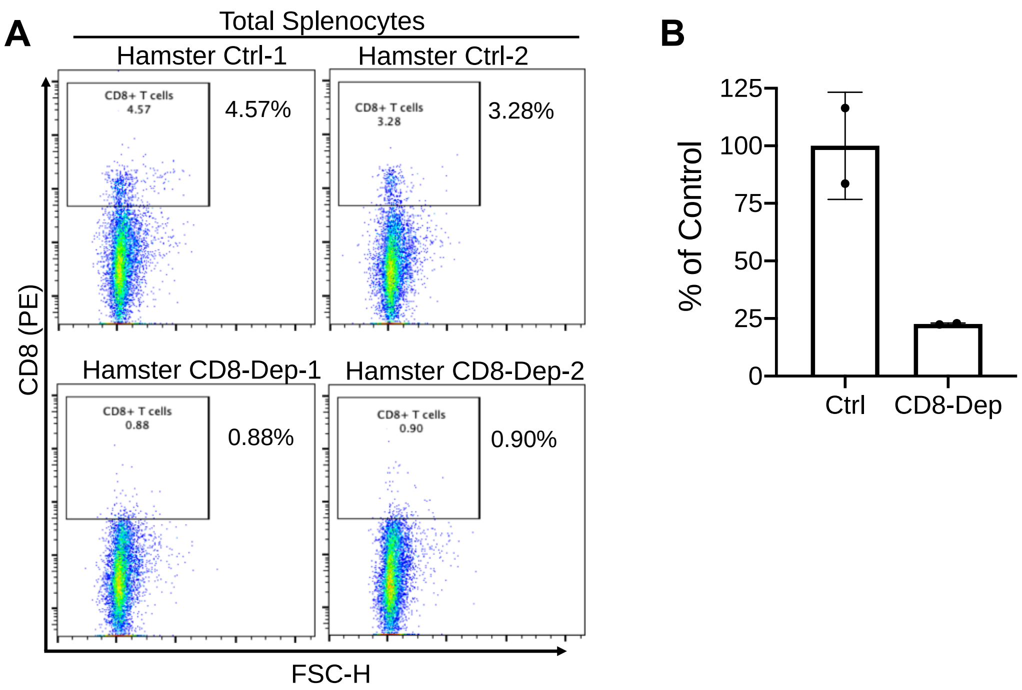

Fig. S6. Confirmation of in vivo CD8$^+$ T cell depletion in hamsters. Hamsters were intraperitoneally injected with either mouse anti-Rat CD8β antibody (175 μg, eBio341, functional grade) or PBS as control on Day -6 and Day -3, as described in the Methods. Three days after second antibody injection (Day 0), splenocytes were isolated from the hamsters and stained with anti-CD8β-phycoerythrin (PE) (clone: eBio341). The percentage of CD8$^+$ T cells in splenocytes was examined by flow cytometry. **(A)** Shown are representative flow cytometry plots for CD8 staining in splenocytes of two control (Ctrl, top) and two CD8-Depleted (CD8-Dep, bottom) hamsters. % CD8$^+$ (or CD8^{hi}) in splenocytes are shown. **(B)** Depletion efficiency was expressed as % CD8$^+$ T cells in splenocytes of the depleted hamsters relative to that of control hamsters (77% depletion). Data are presented as mean ± SD.

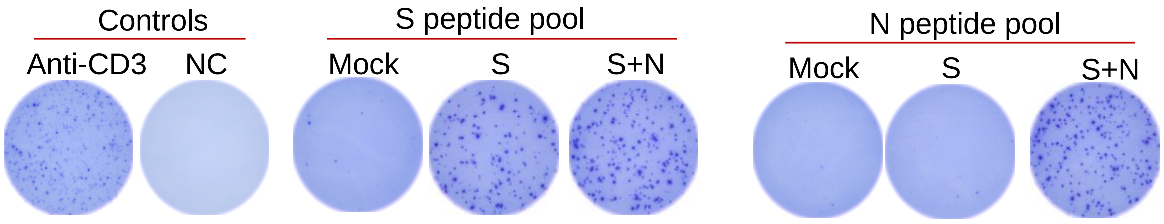

Fig. S7. Immune analysis of mRNA-S and combination mRNA-S+N vaccination in mice. Shown are representative IFN-γ ELISPOT for detecting S-specific and N-specific T cells in the mouse spleen following mock, mRNA-S, or combination mRNA-S+N vaccination (as described in Fig. 5). Positive control (anti-CD3 stimulation) and negative control (medium only) for the IFN-γ ELISPOT are shown.

Data file S1. Raw, individual-level data for all experiments.
